# The second clinical study investigating the surgical method for the kineticomyographic control implementation of the bionic hand

**DOI:** 10.1038/s41598-023-45578-2

**Published:** 2023-10-26

**Authors:** Mahla Daliri, Alireza Akbarzadeh, Behzad Aminzadeh, Amir R. Kachooei, Ghazaleh Hajiaghajani, Mohammad H. Ebrahimzadeh, Ali Moradi

**Affiliations:** 1 Orthopedics Research Center, Ghaem Hospital, Mashhad University of Medical Sciences, Mashhad, Iran; 2 Mechanical Engineering Department, Ferdowsi University of Mashhad, Mashhad, Iran; 3 Department of Radiology, Faculty of Medicine, Mashhad University of Medical Sciences, Mashhad, Iran; 4 Rothman Orthopaedic Institute, Orlando, FL USA

**Keywords:** Mechanical engineering, Orthopaedics

## Abstract

In 2018, during our first clinical study on the kineticomyographic (KMG)-controlled bionic hand, we implanted three magnetic tags inside the musculotendinous junction of three paired extensor-flexor transferred tendons. However, the post-operative tissue adhesions affected the independent movements of the implanted tags and consequently the distinct patterns of the obtained signals. To overcome this issue, we modified our surgical procedure from a one-stage tendon transfer to a two-stage. During the first surgery, we created three tunnels using silicon rods for the smooth tendon gliding. In the second stage, we transferred the same three pairs of the forearm agonist–antagonist tendons through the tunnels and implanted the magnetic tags inside the musculotendinous junction. Compared to our prior clinical investigation, fluoroscopy and ultrasound evaluations revealed that the surgical modification in the current study yielded more pronounced independent movements in two specific magnetic tags associated with fingers (maximum 5.7 mm in the first trial vs. 28 mm in the recent trial with grasp and release) and thumb (maximum 3.2 mm in the first trial vs. 9 mm in the current trial with thumb flexion–extension). Furthermore, we observed that utilizing the flexor digitorum superficialis (FDS) tendons for the flexor component in finger and thumb tendon transfer resulted in more independent movements of the implanted tags, compared with the flexor digitorum profundus (FDP) in the prior research. This study can help us plan for our future five-channel bionic limb design by identifying the gestures with the most significant independent tag displacement.

## Introduction

The number of people in the USA with amputation is currently 2.1 million, and it is predicted to double by 2050, possibly due to increased life expectancy^1^. Upper limb amputation gives rise to various physical and psychological challenges^2,3^. While proximal amputations (at the hip or shoulder) have decreased over time, distal amputations have increased relatively^4^. Recognizing the wearer's intentions is necessary to control bionic limbs. However, obtaining optimized signals for control poses challenges like noise suppression, accommodating a wide range of motion, and ensuring high resolution and fidelity^5^. Electromyography (EMG)-based approaches are considered promising for sensing limb gestures as they generate signals close to the source (muscle belly) and are easier to characterize^6^. However, current EMG-controlled procedures have low identification rates of forearm extrinsic muscles when replicating hand postures, as well as operational issues, often resulting in patient dissatisfaction^7^. Surface EMG (sEMG) is limited because the target muscle is deep inside the limb tissues, making the independent isolation of muscle signals difficult. The predominant EMG technique is direct control, where an electrode is positioned on the skin over two agonist–antagonist muscles^8^. A standard myoelectric hand prosthesis comprises a movable thumb, index finger, and middle finger capable of opening and closing to execute just one grip, specifically the tripod pinch. In contrast, a multi-grip myoelectric hand prosthesis possesses five articulated fingers that can be moved, generating various grips, such as pointing with the index finger, pinching, and gripping objects like a key. This enhanced repertoire of grips offered by the multi-grip myoelectric hand prosthesis often benefits users from improved dexterity. However, adopting multi-grip prostheses has also brought several drawbacks, including increased susceptibility to damage, elevated costs, and intricate and laborious control mechanisms^9,10^. The necessity of employing triggers to switch between different grips is frequently perceived as demanding in terms of time and cognitive effort^10^. Notably, studies have indicated that multi-grip myoelectric hand prostheses did not exhibit significant variations in outcomes compared to standard myoelectric hand prostheses across any of the categories within the International Classification of Functioning, Disability, and Health (ICF)^11^ categories^12^. Implanting muscle electrodes for intramuscular electromyography (EMG) has been explored as a solution and yields enhanced signal quality, yet requires complex surgery and can be prone to injury or inconsistent performance over time^13–15^. Moreover, both invasive and non-invasive EMG approaches solely capture muscle activation and cannot accurately monitor, comprehend, or effectively utilize muscle actions due to insufficient information regarding muscle length and velocity^16^.

To address these challenges, the authors proposed magnet implantation at the musculotendinous junction and detecting magnetic field signals generated during muscle contraction in amputees^17^. Real-time muscle force information, including muscular fibers' length and activation speed, is crucial to determining intended movements accurately. Recent research at MIT introduced a similar Magnetomicrometry method, which utilizes magnetic tags for real-time and wireless muscle force tracking with submillimeter accuracy^18,19^. This innovation facilitates an intuitive and reflexive control mechanism for prostheses and exoskeletons. The MIT researchers conducted a series of experiments^20^ and animal studies^21,22^ centered around the Magnetomicrometry approach. They also explored the surgical implementation of agonist–antagonist myoneural interfaces (AMIs) to incorporate this method for lower limb amputees^23–25^. However, there is a gap in the existing literature concerning the surgical procedure required to apply this method to upper extremity amputations. Given the intricacies of the motor system in the upper extremities, our team has dedicated efforts to develop and present a surgical procedure for implanting magnets through clinical research. Specifically, our proposed approach involves a surgical technique for transferring flexor–extensor tendons to embed the magnetic tags. This procedure is combined with artificial neural networks designed to directly detect human intention from the magnetic fields of the implanted magnets, referred to as KineticoMyoGraphy (KMG) signal. Our first clinical study compared the obtained signal in the Kineticomyography (KMG) method with EMG. We observed that the signal-to-noise ratio was notably higher in KMG signals (-19.5 dB for EMG vs. 4.17 dB for KMG)^26^.

During our first clinical case study, we introduced tendon transfer operation for magnetic tag implantation in the forearm^26^. Although we could recognize hand gestures and grade with high accuracy, we noticed some tissue adhesions lowering tags' independent movement and thus the signals’ distinct patterns with different movements. To solve the problem, we modified our operation method into a two-stage surgery, similar to a conventional two-stage hand flexor tendon repair operation using Hunter rods^27^, but this time on the distal forearm level. Silicon rod causes mesothelial cells to grow around the rod, creating a tunnel between the dorsal and volar aspects of the forearm by developing a highly vascularized pseudo-synovial sheath^28,29^. Silicon rod reduces the chance of adhesions brought on by fibrosis throughout the healing process and supplies the tendon grafts with a great glide surface^30,31^.

The KMG method relies on the movement of magnetic tags and detecting the unique distinct magnetic field signals they generate. Thus, the main determining factor for the accuracy of this control method is the independent movement of the muscle and its corresponding tag. There is currently no known operational technique for implanting magnetic tags into the remaining muscles of the forearm to control bionic limbs^32^. This study aims to apply the modified two-stage surgery for implanting magnetic tags on a trans-radial amputee. This aims to evaluate the displacement of the tags and enhance the robustness of the KMG (kinetic myoelectric signal) controlling system. The tags are implanted in two steps using silicon rods at the musculotendinous junction of the transferred agonist–antagonist muscles in the forearm. These muscles are associated with the thumb, 2nd to 5th finger, and wrist movements.

## Methods

### Set up and ethics

It is an interdisciplinary study between the Orthopedic Research Center at Mashhad University of Medical Sciences and the Center of Advanced Rehabilitation and Robotics Research (FUM CARE) at Ferdowsi University of Mashhad (FUM). This project has received approval from Mashhad University of Medical Sciences' Research Ethics Committee (approval No: IR.MUMS.MEDICAL.REC.1402.113) and is conducted in complete accordance with the Declaration of Helsinki's 1964 ethical conduct (Attachment 1).

### Patient selection

Our inclusion criteria for the amputee tag-reader surgery consist of a young adult patient (18–40 years old) with a distal forearm to wrist amputation that occurred between 3 to 12 months before the study across three trauma centers, demonstrating strong motivation for a bionic hand. Conversely, we have established specific exclusion criteria, such as proximal tissue injuries caused by crushing, infections at the surgical site, other limb disabilities or deformities, any brain or symatic disease or condition with poor upper limbs intentional control, a limited range of motion in shoulder and elbow, diabetes, kidney dysfunction, extensive skin scar, amputations secondary to a tumor, and significant psychological disorders. We utilized our Hospital Information System (HIS) to identify potential candidates for the tag-reader procedure. We searched using the S58.119A ICD-10 code for complete traumatic amputation at the level between the elbow and wrist. This search yielded nine patients who met our inclusion and exclusion criteria. Upon reviewing the records of these selected patients, we found three individuals, aged 20 to 40, who had undergone wrist or distal forearm amputations within the past year. After conducting face-to-face interviews, we successfully recruited a 27-year-old man who had undergone radiocarpal level amputation of his right forearm 11 months prior to the study.

### Pre-operation

The design, manufacturing process, and surface treatment of the magnetic tags followed the specifications outlined in our previous publication^26^. Prior to any intervention, we obtained the patient's informed consent. A physical examination of the affected limb was conducted to assess the remaining muscles’ contraction and function. To ensure the absence of fatty tissue changes in the muscles and to confirm a normal viable neuromuscular condition, bilateral forearm MRI and electrodiagnostic (EDX) studies were performed. An EDX study was individually conducted for each target muscle, involving the insertion of fine needle electrodes into the standard motor points of each muscle, as outlined in Shapiro's textbook^33^:

- FDS: With the patient's forearm supinated, the needle was inserted just medial to the mid-point between the biceps tendon and the mid-wrist.
- EDC: With the patient's forearm pronated, the needle was inserted three to four fingerbreadths distal to the olecranon, three fingerbreadths above the ulna.
- FCR: With the patient's forearm supinated, the needle was inserted four fingerbreadths distal to the midpoint between the biceps tendon and medial epicondyle on a line to the center of the wrist.
- ECR: With the patient's forearm pronated, the needle was inserted just above the lateral epicondyle.

A radiographic examination of the forearm was done to exclude the presence of foreign bodies and to assess the bone condition. Muscles exhibited normal functionality without signs of atrophy in the EDX study.

The last set of three identical tags consists of cylindrical shapes measuring 13.3 mm in length and 5 mm in diameter, with a weight of 1.1 g. Initially, the magnets are inserted into a sterilized capsule and fixed using laser welding. The capsule undergoes electro-polishing and is ultimately sterilized using plasma rays.

### Operation

We aim to embed three magnetic tags within three sets of flexor–extensor forearm muscles. To achieve this objective, the tendon transfer approach was selected as the preferred surgical method. In our prior patient, we described the tendon transfer operation of high torque muscles and the procedure^26^. However, we modified our technique into a two-stage procedure to avoid post-operative adhesions and alterations of tags' independent motions. During the first stage, using silicon rods, we created three separate tunnels for the pair of agonist–antagonist muscles tendon gliding. Next, during the second stage, tendons were transferred, and intramuscular magnets were implanted.

**Figure 1:**
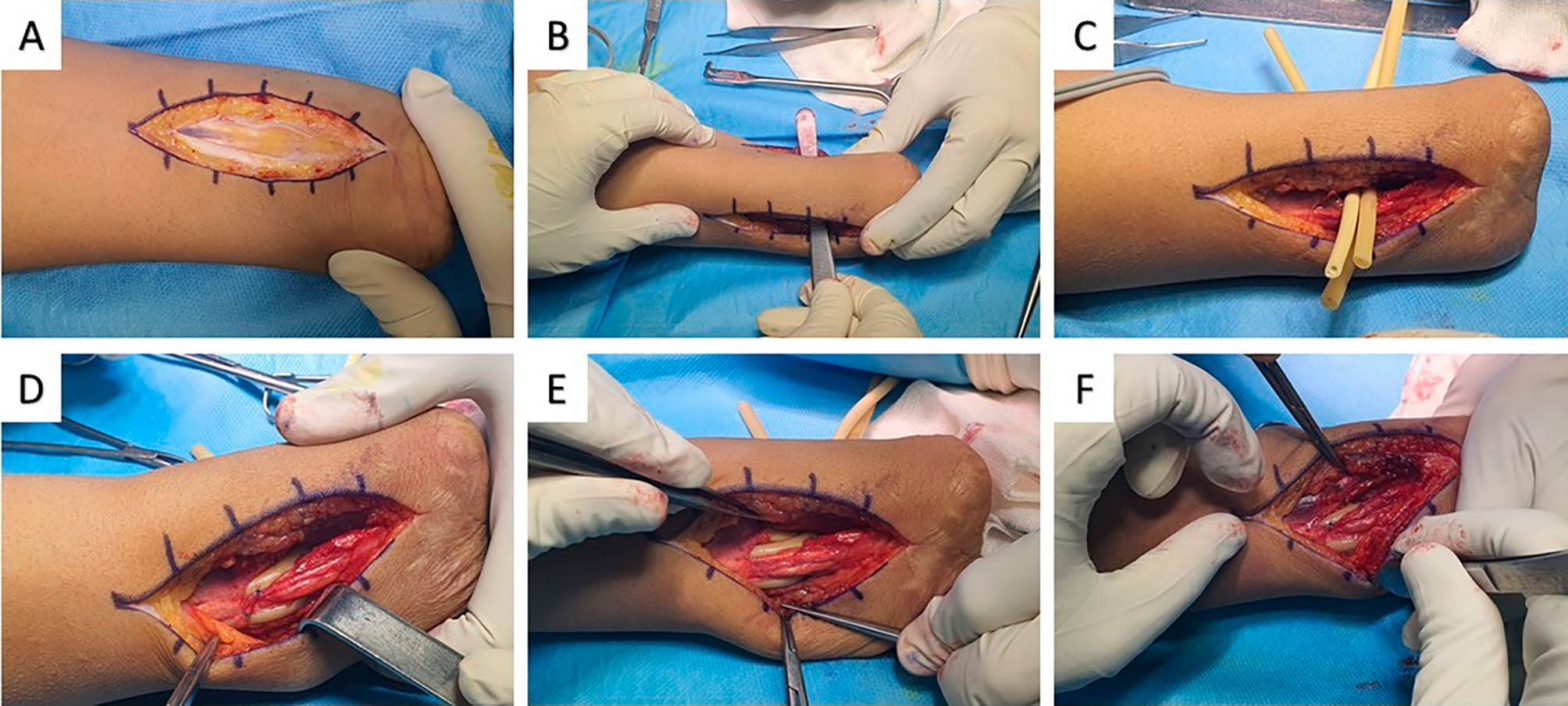
Operation stage one- longitudinal incisions were made on the volar and dorsal aspects of the distal forearm (**A**), and we created three independent volar-dorsal channels through the membrane (**B**). Three 10 mm silicon rods were inserted inside the routs (**C**), and two ends of each rod were fixed to the adjacent soft tissues with a 2–0 nylon suture (**D**, **E**, **F**).

**Figure 2:**
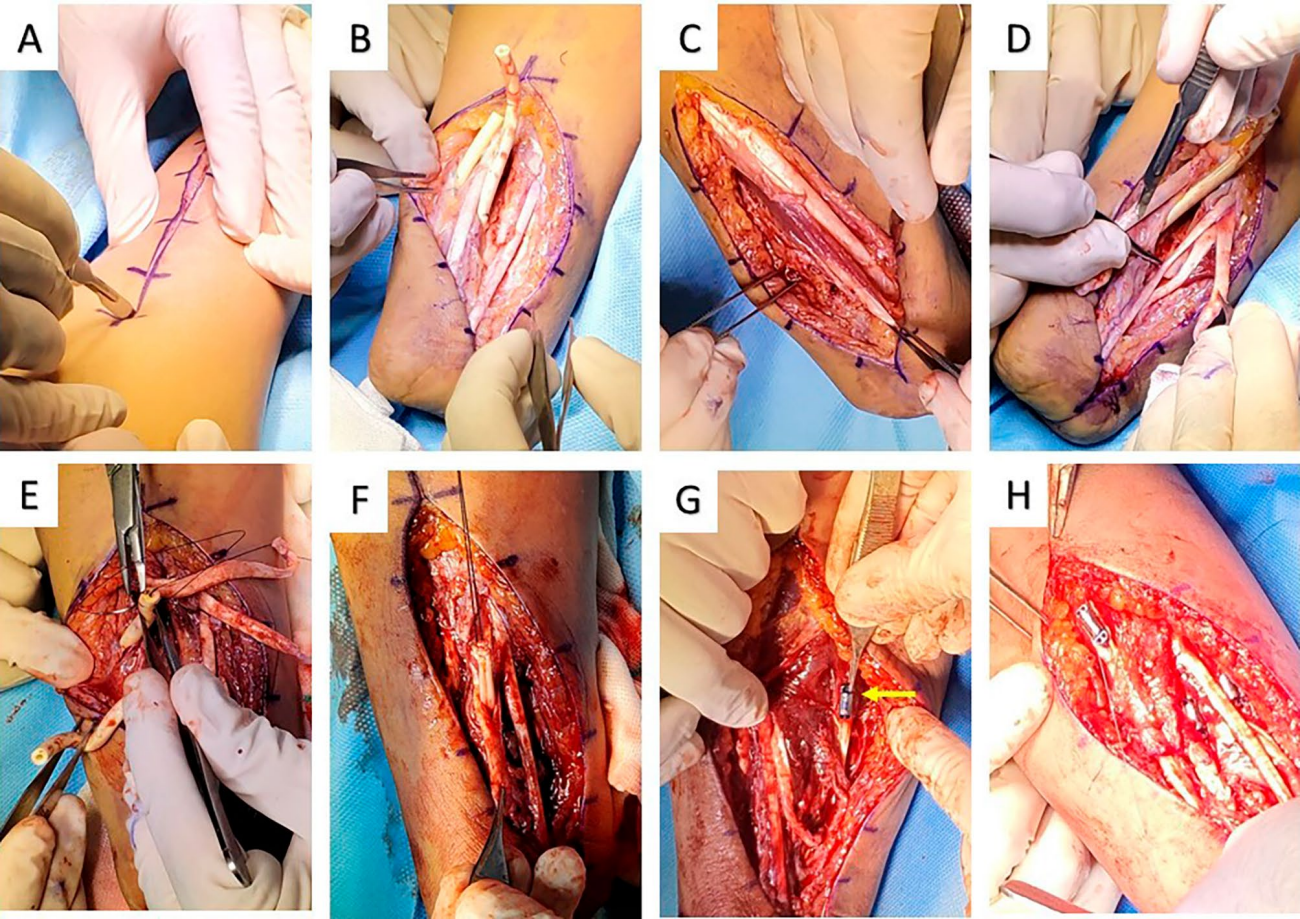
Operation stage two- incisions were made on prior sites on volar and dorsal aspects (**A**). Subcutaneous tissue was dissected, and the inserted silicon rods’ tunnels were determined (**B**). Three pairs of agonist–antagonist tendons were identified (**C**, **D**) and transferred (**E**, **F**). Finally, three magnetic tags were implanted at the musculotendinous junction of volar side muscles (**G** yellow arrow, **H**).

**Figure 3:**
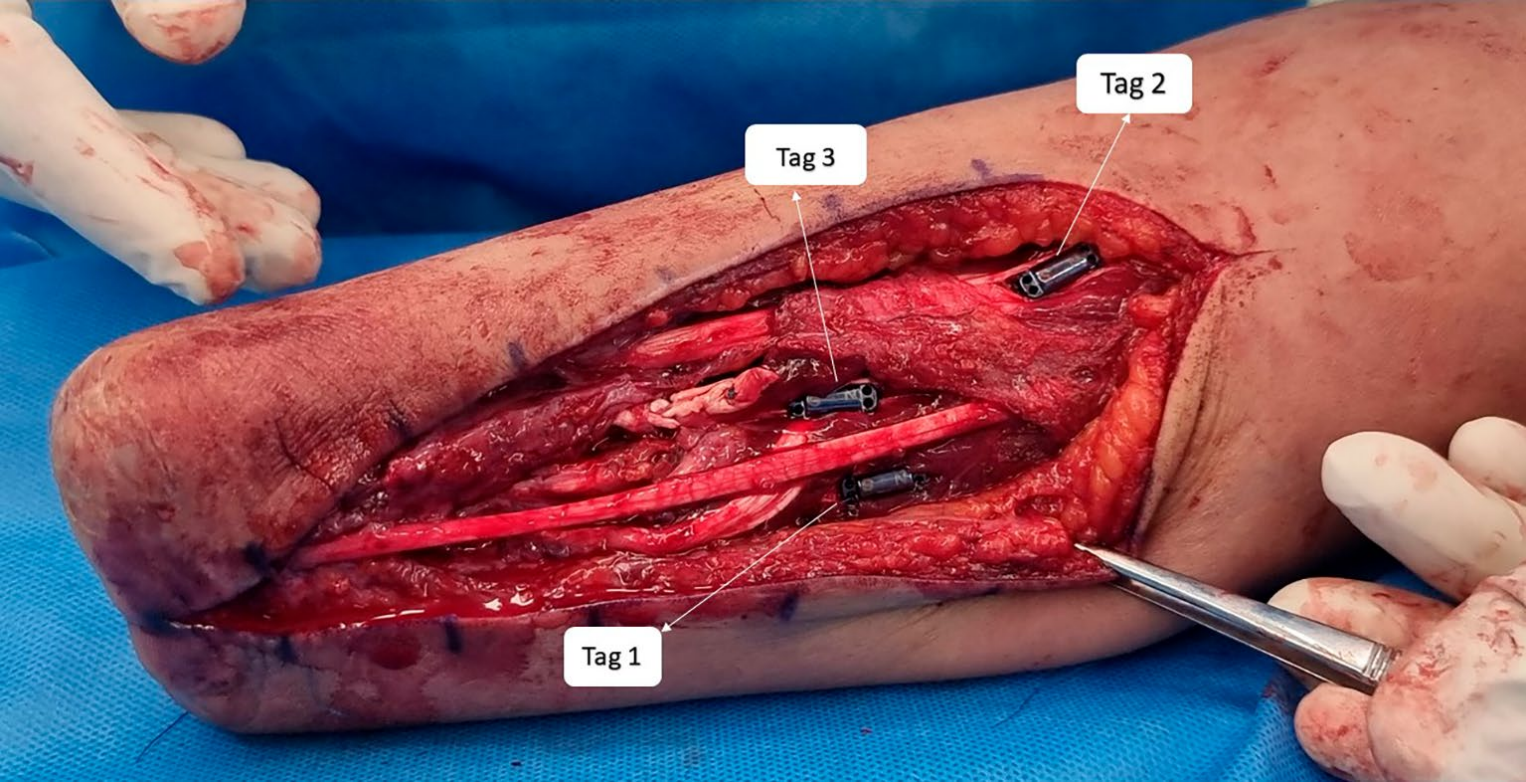
Three magnets implanted in their corresponding agonist–antagonist transferred musculotendinous junction. The ulnar side tag is related to the fingers’ movement (tag 1). The proximal tag is related to wrist movement (Tag 2), and the radial tag is related to thumb movement (Tag 3).

- **Stage 1:** After limb preparation and dressing, the patient underwent general anesthesia. With the patient in a supine position and under tourniquet control, two longitudinal incisions were made on the volar and dorsal aspects of the distal forearm (stump) (Fig. 1A). Through a volar incision, after skin and subcutaneous release, tissue dissection was carried down to reach the interosseous membrane from the radial and ulnar sides. At this stage, we evaluated the muscle bellies and the tendon quality and length of the tendons to plan for the second stage. The muscles were dissected from the interosseous membrane (IOM). On the dorsal aspect, we did the same to reach the interosseous ligament. At the most distal border of the IOL, we created three independent volar to dorsal tunnels (Fig. 1B) through the IOL. Then, three silicon rods 6 mm in diameter were passed through the tunnels (Fig. 1C), and the ends of each rod were fixed to the adjacent soft tissues with a 2–0 nylon suture (Fig. 1D–F). Incisions were irrigated and sutured in layers. A bulky dressing was applied to the forearm.
- **Stage 2:** Six weeks after the first operation, the patient underwent the second stage surgery. Under general anesthesia, incisions were made using the prior scars on the volar and dorsal aspects (Fig. 2A). Subcutaneous tissue was dissected, and the silicon rods’ tunnels were determined (Fig. 2B). An irritating median neuroma in the distal stump was excised and buried deep in the soft tissue. We dissected the soft tissue to find the intact tendons and muscles for three agonist–antagonist tendon transfers (Fig. 2C,D). At the volar aspect, we found Flexor Carpi Radialis (FCR) and Flexor Digitorum Superficialis (FDS) of the 2nd and 4th fingers as the most appropriate intact tendon-muscles for transfer. In the dorsal aspect, Extensor Carpi Radialis Brevis (ECRB), Abductor Pollicis Longus (APL), Extensor Pollicis Brevis (EPB), and Extensor Digitorum Communis (EDC) were found appropriate for transfer. For agonist–antagonist transfer, the sutures at the ends of the silicon rods were removed. Then, according to the tendon length, the longer tendon was attached to the silicon rod to shuttle through the tunnel by pulling the silicon rod out. The agonist–antagonist tendons were attached using a Pulvertaft suture (Fig. 2E,F). We completed three pairs of transfer as follows: Flexor Digitorum Superficialis (FDS) of the fourth finger tendon released and transferred to the Extensor Digitorum Communis (EDC) of the third and fourth fingers on the ulnar side (tag 1). The Flexor Carpi Radialis (FCR) tendon was released on the proximal side and transferred to the Extensor Carpi Radialis Brevis (ECRB) on the radial side (tag 2). Flexor Digitorum Superficialis (FDS) on the distal side related to the second finger tendon released and transferred to the Abductor Pollicis Longus (APL) and Extensor Pollicis Brevis (EPB) tendons (tag 3) (Table 1 and Fig. 3). Finally, three magnetic tags were implanted at the musculotendinous junction on the forearm volar side (Fig. 2G,H). Incisions were irrigated and sutured in layers. A bulky dressing was applied to the forearm.

**Table 1:** Flexor and extensor components of each pair of the transferred tendons.

Component	Tag 1 (fingers)	Tag 2 (wrist)	Tag 3 (thumb)
Flexor component	FDS 4th finger	FCR	FDS 2nd finger
Extensor component	EDC 3rd and 4th fingers	ECRB	APL and EPB

FDS: Flexor digitorum superficialis; EDC: extensor digitorum communis; FCR: flexor carpi radialis; ECRB: extensor carpi radialis brevis; APL: abductor pollicis longus; EPB: extensor pollicis brevis.

### Post-operation

#### Rehabilitation protocol

Sutures were removed after two weeks. The patient underwent a self-rehabilitation program in which he was instructed to perform specific exercises on the intact side while simultaneously simulating the actions on the amputated side. Rehabilitation started the day after the surgery and continued for three weeks. During this period, the patient was directed to perform the following exercises twice a day, completing 10 cycles for each of the movements:

1. Flexion and extension of the wrist
2. Grasp and release of the 2nd to 5th fingers
3. Extension and flexion of the thumb

After this initial phase, the patient was encouraged to incorporate more complex movements into his routine for another three weeks. These included:

1. Independent flexion and extension of the 2nd-5th fingers
2. Supination and pronation of the forearm
3. Cup grip.
4. Tripod pinch
5. Key pinch

After 24 weeks following the surgery, the fluoroscopic evaluation examined the tags’ movements.

#### Fluoroscopic evaluation

To measure the tag displacements, the fluoroscopic images are used to determine the moments when the tags had maximum displacement, which is then compared with the neutral position. The day after surgery, the patient was asked to perform specific hand movements, including finger, thumb, and wrist flexion and extension, while being observed under fluoroscopic imaging to evaluate the positioning of magnetic tags placed during the surgery. At six months follow-up, a second fluoroscopic study was conducted with more accurate measurements in a cardiovascular catheterization lab. Fluoroscopic images were analyzed utilizing the MicroDicom DICOM 2022.2, software. The magnetic tag length of 13.3 mm served as the reference scale, and the extent of displacements was assessed in relation to this tag length (Attachment 2).

#### Ultrasound evaluation

An experienced musculoskeletal radiologist evaluated the displacement of each magnetic tag six months after the surgery using the 12 MHz linear probe from the Samsung WS80 system (Attachment 3). The probe was aligned parallel to the axis of the magnetic tags during all movements, except for the supination/pronation motion, for which the probe was positioned perpendicular to the magnetic tags' axis to measure the tags' mediolateral displacements. Ultrasound evaluation was not conducted on the day immediately after the surgery due to dressing on the forearm, preventing the placement of the ultrasound probe on the skin.

We asked the patient to simultaneously perform the gestures described below with both the amputated and unaffected limbs in three cycles while keeping their eyes closed. Observing the unaffected limb's movements allowed us to assess the gestures' accuracy. Tags motion was assessed using both fluoroscopy and ultrasound with each hand gestures described below:

- Isolated finger flexion and extension: The patient was instructed to isolate each finger and flex and extend the 2nd to 5th fingers individually. Furthermore, the patient was instructed to perform synchronous flexion and extension movements, explicitly involving the 2nd and 3rd fingers, as well as the 4th and 5th fingers, in unison.
- Thumb flexion and extension: The patient was instructed to flex and extend the thumb. Thumb movements are crucial for fine motor tasks such as grasping and pinching.
- Wrist flexion and extension: The patient was instructed to attempt to flex and extend the wrist. Wrist flexion and extension are important for various activities such as writing, typing, playing sports, and performing daily chores.
- Forearm supination-pronation: With the forearm in a neutral position, the patient was instructed to supinate and pronate the forearm. These movements are essential for activities such as turning a doorknob or using a screwdriver.
- Grasp and release: The patient was instructed to either curl up (flexion) or straighten out (extension) all his fingers and the thumb. The full grip is important for the hand's gross and fine motor control. It requires the activation of multiple muscles in the hand and forearm to provide a stable grip and prevent dropping the object.
- Key pinch: The patient was instructed to use his thumb and index finger to hold an object, such as a key or a pen. This grip requires fine motor control and coordination between the thumb and finger muscles to hold an object securely.
- Tripod pinch: The patient was instructed to use the tips of his thumb, index finger, and middle finger to hold an object, such as a pen or a toothbrush. This grip provides stability and precision for fine motor tasks such as writing or brushing teeth.
- Cup grip: The patient was instructed to wrap their fingers around an imaginary object, such as a cup or a ball. This grip is used for holding larger objects securely and requires the activation of multiple muscles in the hand and forearm to provide a stable grip.

### Ethics approval and consent to participate

Ethical approval for this study was obtained from the Research Ethics Committee of Mashhad University of Medical Sciences, Mashhad, Iran (approval No: IR.MUMS.MEDICAL.REC.1402.113). This research was conducted in full compliance with the codes of ethical conduct from the 1964 Declaration of Helsinki. The patient was provided with a written informed consent form before he was enrolled.

### Statement of the location

The present study was performed in Imam Reza Hospital, Mashhad University of Medical Sciences (MUMS).

## Results

### Fluoroscopy

**Figure 4:**
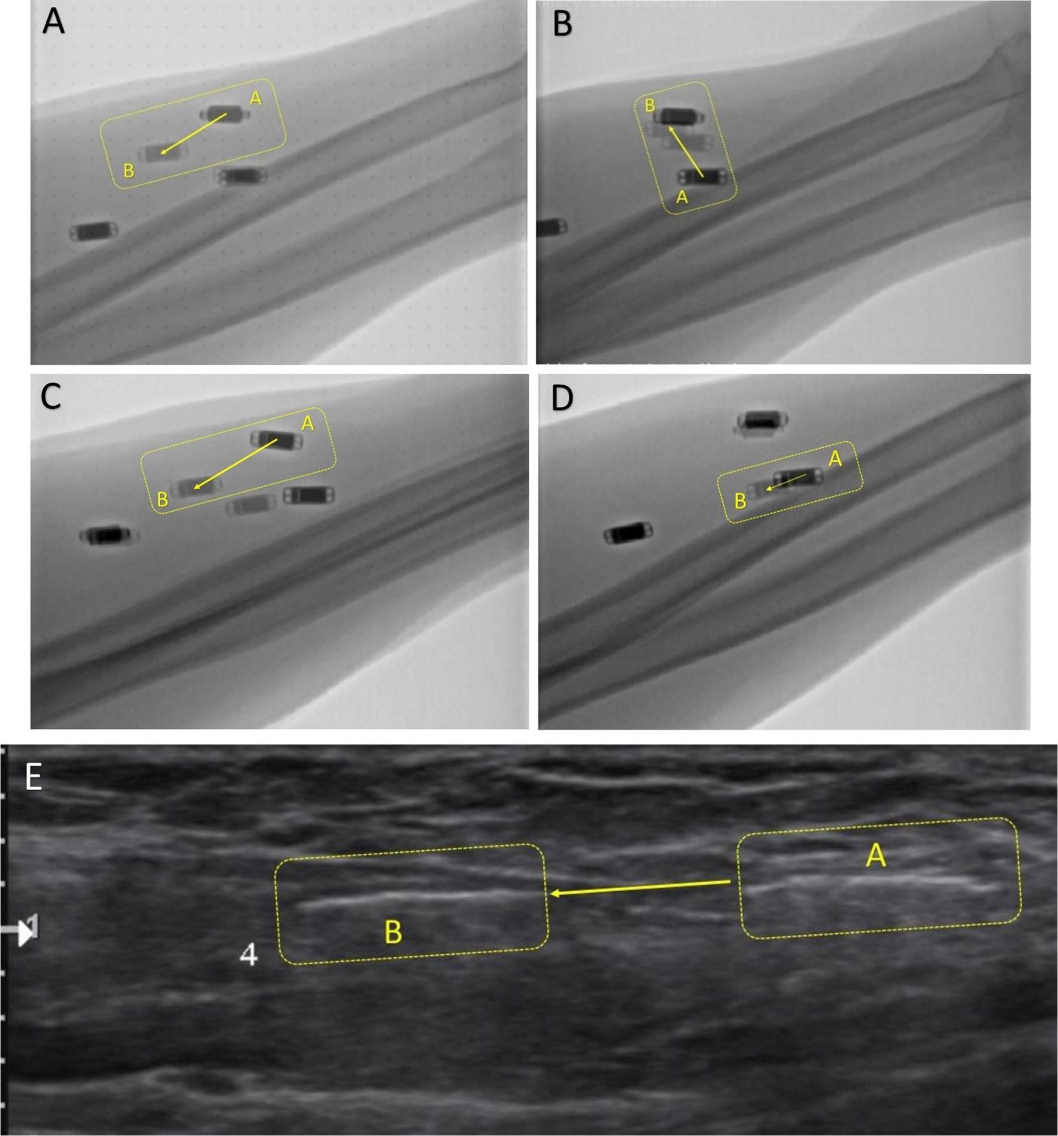
Some of the independent tags’ movements in fluoroscopy and ultrasound: Tag 1 displacement with 4th and 5th fingers Flex-Ext in fluoroscopy (**A**), Tag 3 displacement with the forearm supination-pronation in fluoroscopy (**B**), Tag 1 displacement with grasp and release in fluoroscopy (**C**), Tag 3 displacement with 2nd finger Flex-Ext in fluoroscopy (**D**); Tag 1 displacement with 4th and 5th fingers Flex-Ext in ultrasound (**E**).

**Figure 5:**
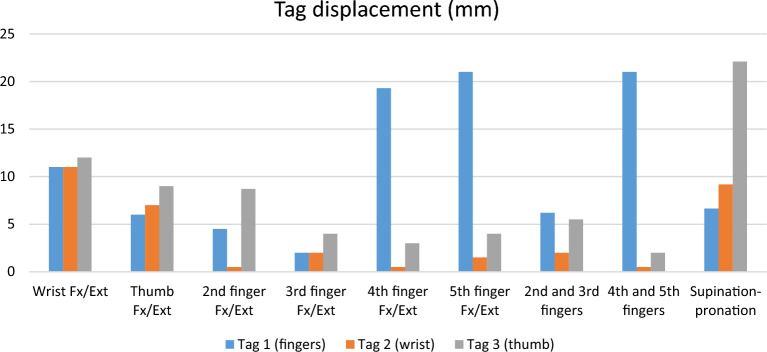
Displacement of the three tags for each simple hand movement in fluoroscopy.

**Figure 6:**
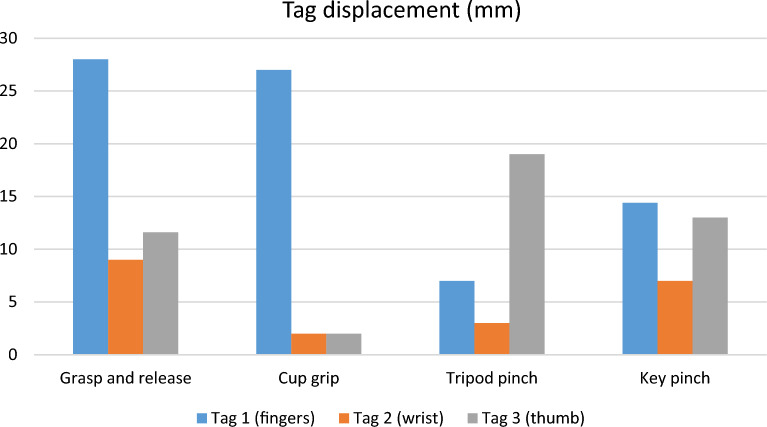
Displacement of the three tags for each complex hand movement in fluoroscopy.

The FDS to EDC tag (tag 1) displacement was 20 mm during grasp-and-release one day after surgery. The FDS to APL and EPB tag (tag 3) displacement was about 5 mm with thumb flexion and extension. The FCR to ECRB tag (tag 2) displacement was minimal without independent movement (Table 2). At six months follow-up, the tags displacement increased, generally. The tag 1, responsible for fingers’ flexion–extension, showed the independent displacement in the following movements: 4th finger, 5th finger, 4th and 5th finger flexion–extension, grasp and release, and cup grip. The thumb tag was independently displaced with forearm supination/pronation, tripod pinch, and the 2nd finger flexion–extension (Table 2, Figs. 4A-D, 5, and 6).

**Table 2:** Tag displacement in fluoroscopy (mm).

Movement	Tag 1 (fingers)	Tag 2 (wrist)	Tag 3 (thumb)
**One day after surgery**			
Grasp and release	**20**	0	4
Wrist Fx/Ext	10	0	7
Thumb Fx/Ext	0	0	**5**
**6-month F/U after surgery**			
Grasp and release	**28**	9	11.6
Wrist Fx/Ext	11	11	12
Thumb Fx/Ext	6	7	**9**
2nd finger	4.5	0	**8.7**
3rd finger	2	2	4
4th finger	**19.3**	0	3
5th finger Fx/Ext	**21**	1.5	4
2nd and 3rd fingers	6.2	2	5.5
4th and 5th fingers	**21**	0	2
Cup grip	**27**	2	2
Tripod pinch	7	3	**19**
Key pinch	14.4	7	13
Supination- pronation	6.64	9.19	**22.09**

Fx/Ext: flexion and extension.

Significant values are in bold.

### Ultrasound

**Figure 7:**
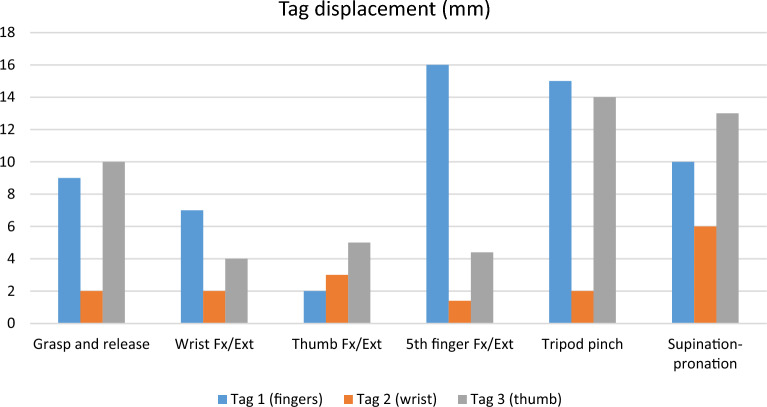
Displacement of the three tags for each hand movement in ultrasound.

An ultrasound evaluation was conducted at six months post-op. The FDS to EDC tag (tag 1) displacement was 16 mm with 5th finger flexion and extension six months after surgery. The FCR to ECRB tag (tag 2) displacement was minimal without independent movement (Table 3 and Figs. 4E and 7).

**Table 3:** Tag displacement (mm) in sonography at six months F/U after surgery.

Movement	Tag 1 (fingers)	Tag 2 (wrist)	Tag 3 (thumb)
Grasp and release	9	2	10
Wrist Fx/Ext	7	2	4
Thumb Fx/Ext	2	3	5
5th finger Fx/Ext	**16**	1.4	4.4
Tripod pinch	**15**	2	**14**
Supination- pronation	10	6	13

Fx/Ext: flexion and extension.

Significant values are in bold.

## Discussion

This trial is the continuum of our earlier investigation on bionic limb KMG control to address the prior limitations on surgical aspects and subsequently improve the control accuracy. To achieve this, we adjusted our surgical method and introduced a two-stage operation resulting in independent tags motion. By identifying the hand gestures in which the optimum independent movements of the tags occur, we can plan for a more robust controlling system with more channels to control more gestures of the bionic hand.

To create an optimized and straightforward electromechanical structure for the hand, it was determined that the three most beneficial hand movements are flexion and extension of the thumb, abduction and adduction of the thumb, and flexion and extension of the 2nd to 5th fingers. These three movements enable 22 different grasping actions and cover 81% of daily tasks^34^. Therefore, it was decided to allocate three actuators (magnetic tags in this study) to control the thumb, fingers, and wrist degrees of freedom in order to achieve optimized control.

Compared to the other two pairs of muscles, the inserted tag in the flexor–extensor tendons of the fingers demonstrates the highest range of motion. In our previous trial, the movement of the tag in the fingers measured 4.4mm and 5.7mm one day after the surgery and at the 3-month follow-up, respectively^26^. However, the present study shows a range of 20mm and 27mm one day and six months after the surgery, respectively. This improvement can be attributed to the change we made in the flexor component of this muscle pair. In our previous trial, we used the flexor digitorum profundus (FDP), while in the current study, we used the flexor digitorum superficialis (FDS) of the 4th finger. The FDS has separate muscle slips for each of the four digits, allowing for more independent control. Additionally, the FDS exhibits higher excursion during active movements of a straight fist, PIP joint block, and DIP joint block, with less force than the FDP^35,36^. The flexion–extension of the 4th and 5th fingers is most associated with the independent movements detected by tag 1.

FDS muscle assists in wrist flexion, which is probably why the observed movement of the FDS tag was notable during wrist flexion–extension (10mm). The tag implanted into the flexor–extensor tendons of the wrist also exhibited increased mobility compared to our previous experiment (measuring 4.7mm vs. 11mm). The adjustment in surgical technique improved the independent movement of this tag by reducing adhesion issues with silicon rods. The tag related to the thumb showed displacements of 9mm, 12mm, and 20mm with thumb, wrist, and finger flexion–extension, respectively. This is because the flexor component in the thumb's implanted tag is the flexor digitorum superficialis (FDS). In the present trial, the third magnetic tag was implanted into the flexor–extensor tendons of the thumb, with the extensor component being the extensor pollicis brevis (EPB) and abductor pollicis longus (APL) tendons instead of the extensor pollicis longus (EPL), in the prior study. We anticipated that having two extensors would significantly contribute to the movement of this tag. Furthermore, EPB has been observed to have an excellent excursion in the neutral position of the wrist^37^. As predicted, there was a noticeable difference in the outcomes between the present study and the previous one regarding the independent movement of the tag corresponding to the thumb (measured 3.2 mm during the last trial versus 9 mm in the present study).

The potential reasons for the inconsistent results between fluoroscopy and ultrasound could be as follows: First, the patient could not replicate the movements with the same level of motion accuracy and muscle contraction strength during different imaging evaluations. This discrepancy in motion replication could be a primary factor leading to differing detection results during each follow-up evaluation. Second, the ultrasound study requires placing the probe directly on the patient's operated skin, which could induce pain. Additionally, the ultrasound detection process is more time-consuming for each tag and movement individually. Consequently, the patient's arm fatigue might result in fewer forceful movements and, subsequently, fewer tag displacements. Third, fluoroscopy involves a magnification effect that complicates the assessment of the extent of this effect. While this might be a minor consideration, it could contribute to the observed discrepancies.

In our proposed surgical approach, we avoided the confounding effect of axial and radial tag displacement by inserting magnetic tags within the musculotendinous junction rather than inside the muscle mass. To achieve accurate measurement of muscle length, Herr et al.^18^ recommended the implantation of multiple magnets per muscle, which introduces a more complex surgical procedure. However, this challenge can also be addressed by utilizing an intraosseous tag as the reference point for the muscular tags. Additionally, when tags are implanted deeper within the muscle mass than the musculotendinous junction, accurate tracking is limited by sensor noise from the magnetic field sensors^38^.

Limitations: We did not measure the displacement of the magnets in relation to the suture positions precisely. However, the tags remained in the exact location in our serial radiographic follow-up with the forearm in a neutral position. For future studies, we recommend utilizing sonography prior to the surgery to actively identify the most suitable muscles with independent movement for transfer.

## Conclusion

Compared to our initial clinical study, where independent movements were achieved in only one magnetic tag, two-stage surgical modification implemented in the current study resulted in more significant independent movements observed in two magnetic tags, specifically related to the 4th and 5th fingers and thumb flexion–extension. Furthermore, we observed that FDS tendons result in more independent tag movements compared with the FDP in the prior research. Moreover, finger tendon agonist–antagonist tendon transfer has a higher chance of independent movement than wrist tendons.

### Supplementary Information

Supplementary Video 1.

Supplementary Video 2.

Supplementary Video 3.

Supplementary Video 4.

Supplementary Video 5.

Supplementary Video 6.

Supplementary Video 7.

Supplementary Video 8.

## Data Availability

Correspondence and requests for materials should be addressed to almor0012@gmail.com.
